# Impact of Pelvic Bone Anatomy on Inguinal Hernia and the Role of Radiological Pelvimetry

**DOI:** 10.7759/cureus.21269

**Published:** 2022-01-15

**Authors:** Akash Rajeev, Ashwin Vinod, George M John, Pradeep Jacob, Riju Ramachandran, Vishal Marwaha

**Affiliations:** 1 General Surgery, Amrita Institute of Medical Sciences and Hospital, Kochi, IND; 2 Rheumatology, Amrita Institute of Medical Sciences and Hospital, Kochi, IND

**Keywords:** x-ray pelvis, inguinal hernia repair, pelvimetry, groin hernia, pelvic anatomy

## Abstract

Introduction

One of the most prevalent disorders treated by general surgeons is inguinal hernias. Many of the etiological factors that lead to hernia development are unknown. This study looked at the role of pelvic bone anatomy in the development of inguinal hernia. The pubic tubercle's location (as measured by the Radoievitch angle) and its relationship to the formation of inguinal hernia, as well as its function in the pathophysiology of various forms of inguinal hernia, were investigated.

Materials and methods

From October 2019 to June 2021, a prospective case-control study with 70 individuals over the age of 18 years in each arm was conducted in the Department of General Surgery at our tertiary care institution. Plain digital X-ray radiography of the pelvis, including bilateral hips, was taken in the anteroposterior (AP) view with the patient in the supine position, and the Radoievitch angle and Ami line were measured using suitable measuring instruments. The required information for patients in both groups was tallied and examined in a data sheet.

Results

Between the case and control groups, there was a statistically significant difference in the mean Radoievitch angles and the mean length of the Ami line (42.46 +/-2.442 degrees vs 40.91 +/-2.547 degrees; p<0.05); (8.54+/-1.059 cm vs 7.27+/-1.034 cm; p<0.05). There was a statistically significant increase in the Radoievitch angle of patients with bilateral hernias compared to unilateral hernias (p-value <0.01), as well as indirect hernias compared to other forms of hernias (p-value <0.05).

Conclusion

The probability of having an inguinal hernia was higher when the Fruchaud region was increased, as indicated by a larger Radoievitch angle or a longer Ami line. A low-lying pubic tubercle increased the likelihood of bilateral and indirect inguinal hernias. Pelvimetry is an easy test that should be considered on a routine basis and can be applied accurately in all patients. Surgeons can employ pelvimetry to identify patients who are more likely to benefit from non-mesh repairs.

## Introduction

Hernia by definition is the abnormal protrusion of any viscus or peritoneum lined sac outside of its normal containing cavity through some natural or unnatural opening [1].

Among the most prevalent disorders treated by general surgeons are inguinal hernias. All the etiological factors that lead to hernia development are unknown. According to Fruchaud, all groin hernias start in a single weak location termed the myopectineal orifice [1]. The absence of evolutionary development of a robust posterior rectus sheath and transversalis fascia in the lower abdomen is regarded to be a key anatomical flaw in human evolution [2]. Hernia repair success depends on permanent closure of the defect, minimal complications, low expenses, and a quick return to regular activities. The success rate of every surgery is determined by the operating surgeon's comprehension of the anatomy of the operative region and the most effective application of expertise.

The Radoievitch angle, established between the interspinal line uniting the two anterior superior iliac spines and Malgaigne's line on an X-ray pelvis anteroposterior view, was used to determine the position of the pubic tubercle from the interspinal line of the pelvis (the relationship between hernia development and the line connecting the pubic tubercle to the ipsilateral anterior superior iliac spine) [3].

## Materials and methods

From October 1, 2019, to June 30, 2021, a prospective case-control study was conducted in the departments of General Surgery and Gastrointestinal Surgery at our tertiary care hospital in South India.

The case population consisted of patients over the age of 18 years with a clinical diagnosis of inguinal hernia. Individuals without an inguinal hernia who visited the hospital's departments of Emergency Medicine and Orthopedics for treatment of illnesses other than inguinal hernia and who had a pelvic X-ray done for assessment of other ailments were included in the control group. People who had a pelvic fracture or injury, or any other disease that changed the normal structure of the hip bone, or who had had any abdominal procedures other than prior inguinal hernia repairs, were excluded from the research.

**Figure 1:**
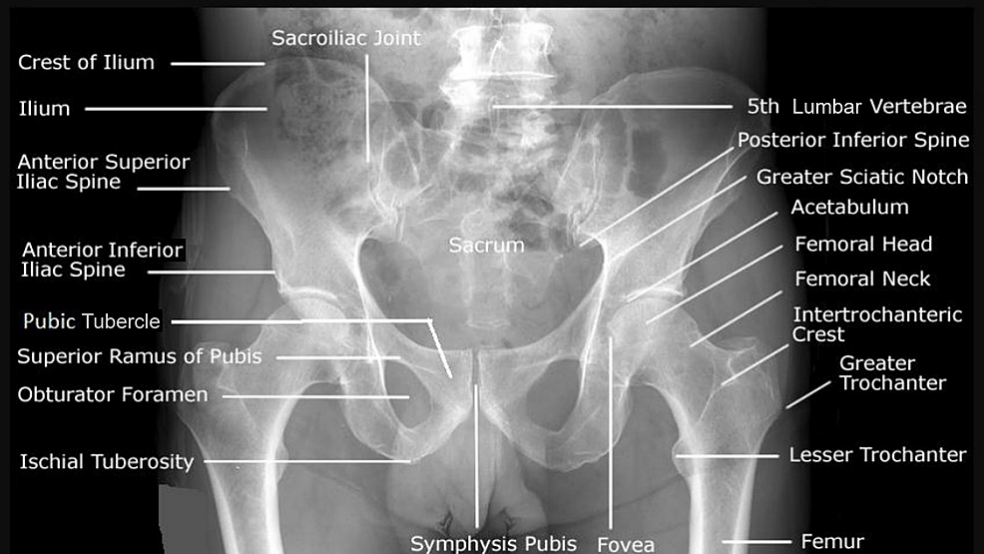
Plain Radiograph of the Pelvis with Bilateral Hip Depicting Bony Landmarks

In all groups, plain digital X-ray radiography of the pelvis, including bilateral hips, was taken in the anteroposterior (AP) view with the patient in the supine position. The Radoievitch angle in degrees and the length of Ami's line in centimetres were measured using suitable measuring tools contained in the programme after seeing X-ray images (Figure 1) in our Hospital Information System Medvision software (Allied Softech Pvt. Ltd., Pune, India).

**Figure 2:**
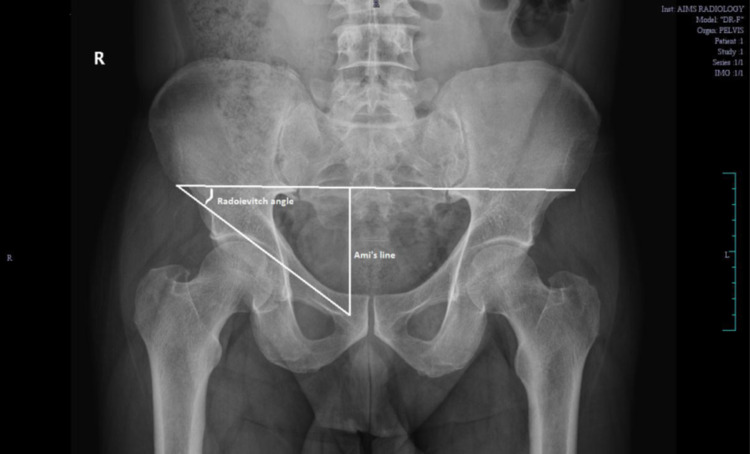
Plain Radiograph of the Pelvis with Bilateral Hip Depicting Radoievitch's angle and Ami's Line

The angle produced by the interspinal line connecting the two anterior superior iliac spines and Malgaigne's line (the line connecting the pubic tubercle to the ipsilateral anterior superior iliac spine) is known as the Radoievitch angle [3]. The perpendicular line between the interspinal line that connects the two anterior superior iliac spines and the pubic tubercle is known as Ami's line (Figure 2) [3].

The information of the study population acquired was based on an examination of medical records, which was then compiled on a data sheet. IBM SPSS Statistics 20 for Windows was used for statistical analysis (SPSS Statistics, Armonk, NY). For all continuous variables, the findings were presented in mean ± standard deviation (SD), while for categorical variables, the results were given in frequency (%). In order to analyse the means of continuous parameters between two groups, Student's independent samples t-test was used. A p-value of less than 0.05 was considered statistically significant.

## Results

During the research period, 70 people were randomly assigned to the case and control arms each. The mean age of the case group was 54.7+/-13.4 years whereas the mean of the control population was 24 50.11+/- 16.6 years. There were 62 males and eight females in the case group with similar distribution in the control group also. Hence both the arms were equally matched with respect to age and gender.

The mean Radoievitch angle in patients with hernia in the case group was 42.46 +/-2.442 degrees, while it was 40.91 +/-2.547 degrees in the control group. Between the two groups, there was a statistically significant difference in mean Radoievitch angles (p < 0.05). The average length of Ami's line in individuals with hernia was 8.54+/-1.059 cm, while it was 7.27+/-1.034 cm in those without hernia. A statistically significant difference (p < 0.05) was found between the two populations (Table 1).

**Table 1:** Comparison of Radoievitch's Angle and Length of Ami's Line Between the Study Groups

Group	n	Mean	Standard Deviation	p value
Radoievitch Angle				
With Hernia	70	42.26	2.442	0.002
Without Hernia	70	40.91	2.547	
Ami's Line				
With Hernia	70	8.54	1.059	<0.001
Without Hernia	70	7.27	1.034	

The mean Radoievitch angle in men with hernia was 42.52+/- 2.414 degrees and in women with hernia the same was 40.25+/-1.669. In the control population, the mean Radoievitch angle was found to be 41.16 +/- 2.556 degrees in men while it was 39 +/- 1.512 degrees in women. The difference in the mean Radoievitch angles in the case arm and the control arm respectively were statistically significant in both men and women. The mean length of Ami’s line in men with hernia was 8.65+/- 0.993 cm and in women with hernia it was 7.75+/-1.282 cm. The mean length of Ami’s line in men without hernia was 7.31+/-1.065 cm and in women without hernia was 7.00+/- 0.756 cm. There was a statistically significant difference in the mean length of Ami's line between male and female individuals in the case group, but no such difference was found between male and female individuals in the control population (Table 2).

**Table 2:** Comparison of Radoievitch’s Angle and Length of Ami’s Line Between the Study Groups Based on Gender

Group	Gender	n	Mean	Standard Deviation	p-value
Radoievitch Angle					
Control Population	Female	8	39.01	1.512	0.023
	Male	62	41.16	2.556	
Case Population	Female	8	40.25	1.669	0.012
	Male	62	42.52	2.414	
Ami's Line					
Control Population	Female	8	7.01	0.756	0.434
	Male	62	7.31	1.065	
Case Population	Female	8	7.75	1.282	0.023
	Male	62	8.65	0.993	

The case population was divided as right, left, or bilateral based on the side of occurrence of hernia. The Radoievitch angle was statistically significantly higher in people with bilateral hernias than in people with unilateral hernias (p-value < 0.05) (Table 3).

**Table 3:** Comparison of Measurement of Radoievitch’s Angle for Hernia Categorized as Unilateral or Bilateral

Side of Hernia	n	Mean	Standard Deviation	p-value
Radoievitch Angle				
Unilateral	57	41.95	2.386	0.025
Bilateral	13	43.62	2.293	

Based on the intraoperative findings or clinical examination findings, hernias were classified as direct, indirect, or Pantaloon, i.e., a combination of both. When patients with indirect hernia were compared to the rest of the case population, there was a statistically significant difference in the mean measurement of Radoievitch's angle (p-value < 0.05) (Table 4).

**Table 4:** Comparison of Mean Radoievitch’s Angle Between Indirect Hernia and The Other Types

Type of Hernia	n	Mean	Standard Deviation	p value
Radoievitch Angle				
Direct and Pantaloon	48	42.75	2.392	0.012
Indirect	22	41.18	2.239	

## Discussion

Inguinal hernia can afflict both men and women, however it is more common in men [4]. In our study, of the seventy individuals in the case population, eight were women (11.42%). This sex distribution was similar in studies quoted in literature [1,5].

Inguinal hernia affects people of all ages; however, it becomes more common as they become older [5]. The average age of the study group was 54.57 years. Because the final structure of the pelvis is thought to be complete by the age of 12-18 years, only those over the age of 18 were included in this study [6,7]. The most frequent form of hernia, regardless of gender, is an indirect inguinal hernia, which is nearly five times more prevalent than a direct inguinal hernia [8,9].

Direct hernias tend to be more common with advancing age [2]. In our study, the overall incidence of direct hernia was 57.14%. Among the men included in our study, 59.67% had direct hernia while 27.41% had the indirect variety. This discrepancy is most likely due to the fact that or study had a limited sample size, compared to data from big epidemiological studies.

The current study showed that 44.28% of persons had a right-sided hernia whereas 37.14% had left-sided hernia and 18.57% had bilateral hernia. Hernias especially that of the indirect variety are more common on the right side. This is thought to be due to the right-side patent processus vaginalis closing at a slower rate than the left [10].

Unlike prior research where confounding variables such as sex and age were not examined and findings were associated, the outcomes of our case-control study were matched for confounding factors of sex and age. Lopez-Cano et al., in their study, noted a significant gender-based variation in the low position of the pubic tubercle within their case population [7]. The above authors have ascertained the fact that in females the pubis is placed higher than in males (mean value of Radoievitch angle or the length of Ami’s line is smaller in females). Rosen et al. conducted cadaveric dissection of the inguinal region in 50 adult specimens, 26 males and 24 females. The distance between the internal ring and the public tubercle was longer in females than in males, according to anatomical differences in both sexes (at an average of 31.86mm in males and 35.68mm in females) [6]. Females’ rectus muscles were also found to be substantially broader. The mean Radoievitch angle and the length of the Ami's line were also substantially varied between the sexes in our investigation. This is one of the probable contributing factors for the lower incidence of inguinal hernia in females. Harissis et al. found no gender-based difference in their study but that may be attributed to their very small sample size [3]. In a study by Agrawat et al., there was no statistically significant association between the length of the Amis’s line and the side of the hernia [11]. In fact, they recorded longer mean lengths in those with a left-sided hernia, unlike in our study where there was a significant difference in the Radoievitch angle between those with a bilateral and unilateral hernia.

None of the publications reviewed thus far have studied the significance of low-lying pubic tubercle with a type of hernia, based on classification as direct, indirect, and Pantaloon. There was a statistically significant difference in the measure of Radoievitch's angle between indirect hernias and the rest of the group in this research.

Current guidelines for inguinal hernia repair advocate mesh repair for all types of inguinal hernia due to excellent results and exceptionally low recurrence rates and a short learning curve for new surgeons [8]. However, postoperative complications like mesh infection, rejection, and poor patient satisfaction due to persistent postoperative pain and infection are causes for worry [7]. Nyhus, in an article published earlier, suggested that mesh repair was not required in Types I and II and IIIC hernias [12]. In view of the low recurrence rate, the Nyhus Type IIIA, IIIB, and IV hernias appear to have a better risk-to-benefit ratio, especially if the mesh was inserted posteriorly, either by open or closed surgeries [7]. A large Fruchaud's area would arise from a low-lying pubic tubercle, therefore a tension-free repair with some form of prosthetic material would be desirable [6,13]. Given the intrinsic stress induced within the tissues when performing a primary repair, any attempt to restore this large posterior part of the inguinal canal without mesh insertion would be more likely to fail.

Pelvimetry, according to Rosen et al. and van Wassem et al., is a very straightforward test that should be utilized on a regular basis [6, 14]. However, Radoievitch's research (and those of others) focused mostly on prepared cadaveric specimens. On the other hand, patient-taken pelvic measures are not always precise, because interference of subcutaneous fat can drastically influence the conclusion of pubic height measurement. The approach used in this study, as well as that used by Harissis et al., using radiological pelvimetry, solved this obstacle and could be used properly in all situations [3,15]. Surgeons might utilize radiological pelvimetry to identify patients who would benefit from non-mesh procedures like the Shouldice technique (small Radoievitch's angle, resulting in a low pubic height and smaller Fruchaud's area).

As mesh repair is now the standard of care for treating inguinal hernias, anatomical repair of these hernias would require additional evidence before being adopted as a standard protocol in the surgical treatment of hernias with low-lying pubic tubercle. As a result, randomized control trials with high sample size and the use of X-ray pelvimetry would give reliable data to distinguish individuals who would benefit from tissue restoration.

## Conclusions

Pelvic bone anatomy, with reference to low lying pubic tubercle, would result in a great Fruchaud’s area (as measured by an increased Radoievitch's angle or an increased length of the Ami line), hence predisposing to the development of an inguinal hernia in both males and females. In addition, the pelvic anatomy varied between men and women with respect to the pubic tubercle. A low-lying pubic tubercle also makes a bilateral inguinal hernia more likely and plays a role in determining which type of inguinal hernia a person would develop later.

Pelvimetry is a very simple examination to be routinely considered. The method of radiological pelvimetry utilized in this study could accurately be applied to all preoperative cases.
